# Preliminary investigation of the *in vitro* anti-*Helicobacter pylori* activity of Triphala

**DOI:** 10.3389/fphar.2024.1438193

**Published:** 2024-11-19

**Authors:** Zhixiang Zhu, Yuanjing Zou, Ling Ou, Meiyun Chen, Yujiang Pang, Hui Li, Yajie Hao, Bingmei Su, Yuqian Lai, Liping Zhang, Junwei Jia, Ruixia Wei, Guimin Zhang, Meicun Yao, Zhong Feng

**Affiliations:** ^1^ School of Medicine and Pharmacy, Ocean University of China, Qingdao, Shandong, China; ^2^ School of Pharmaceutical Sciences (Shenzhen), Sun Yat-sen University, Shenzhen, Guangdong, China; ^3^ Lunan Pharmaceutical Group Co., Ltd., Linyi, Shandong, China; ^4^ Shandong Engineering Research Center for New Drug Pharmaceuticals R&D in Shandong Province, Lunan Better Pharmaceutical Co., Ltd., Linyi, Shandong, China

**Keywords:** Triphala, *Helicobacter pylori*, urease, antimicrobial efficacy, mechanism of action

## Abstract

**Background:**

Triphala, is a composite of three individual botanical drugs: *Terminalia chebula*, *Terminalia bellirica*, and *Emblica officinalis*. It exhibits properties such as heatclearing, anti-inflammatory, anti-fatigue, antioxidant, and antibacterial effects,making it extensively utilized in India and Tibet. It has been found to exhibitinhibitory effects on *Helicobacter pylori* (*H. pylori*); however, further comprehensive research is still needed to elucidate its specific antibacterial mechanism. The present study investigates the in vitro antibacterial activity and antibacterial mechanism of Triphala against *H. pylori*.

**Methods:**

Ours research investigates the *in vitro* inhibitory activity of Triphala on multiple standard and clinical strains using microdilution broth method, time-kill curve, time-bactericidal curve and scanning electron microscopy (SEM). Furthermore, the antibacterial mechanism of Triphala is further explored through experiments on urease activity, biofilm formation, anti-adhesion properties, virulence actor assays using RT-qPCR and Western Blotting techniques.

**Results:**

The research findings indicate that Triphala exhibits a minimum inhibitory concentration of 80–320 μg/mL against both standard and clinical strains of *H. pylori*. Triphala exerts its *anti-H. pylori* effect by perturbing the microstructure of *H. pylori*, downregulating adhesion-associated genes (*alpA, alpB, babA*), urease-related genes (*ureA, ureB, ureE, ureF*), and flagellar genes (*flaA, flaB*); inhibiting bacterial adhesion, biofilm formation, urease activity as well as *CagA* protein expression.

**Discussion:**

These findings suggest that Triphala exerts inhibitory effects on *H. pylori* activity through multiple mechanisms, underscoring its potential as a new drug for the prevention and treatment of *H. pylori* infection.

## 1 Introduction

*Helicobacter pylori* (*H. pylori*), a gram-negative bacterium, exhibits a slender and curved or spiral rod-shaped morphology. It thrives in microaerophilic conditions and possesses flagella at its posterior end. *H. pylori* has coexisted with the human host for millions of years (Hathroubi, Zerebinski, and Ottemann, 2018). However, it was not until the 1980s that researchers successfully isolated *H. pylori* from the gastric samples of patients, thereby obtaining initial insights into its characteristics (Hathroubi, Zerebinski, and Ottemann, 2018; Malfertheiner et al., 2023). *H. pylori* infection affects over half of the global population (Polk and Peek, 2010). Although the majority of cases are asymptomatic, a considerable proportion of patients manifest various gastrointestinal disorders consequent to their infection, including chronic gastritis, gastric ulcers, and duodenal inflammation. Moreover, it is also deemed to be associated with the incidence of stomach cancer (Backert et al., 2016). In the 2015 Kyoto Conference on *H. pylori* gastritis, *H. pylori* was regarded as a highly pathogenic bacterium that requires eradication in all infected people (Sugano et al., 2015). The International Agency for Research on Cancer has classified it as a Group 1 carcinogen, indicating its high potential to cause gastric cancer (Ansari and Yamaoka, 2022). Approximately 10%–15% of individuals infected with *H. pylori* will develop peptic ulcers, and among them, 1%–2% of *H. pylori*-infected patients will progress to gastric cancer (Uemura et al., 2001; Wang, 2014). Therefore, the presence of *H. pylori* infection poses a significant threat to human health.

Currently, the predominant therapeutic regimens for *H. pylori* infection worldwide encompass triple therapy involving a combination of antibiotics (two distinct types) and proton pump inhibitors, as well as quadruple therapy incorporating bismuth-containing agents. Although these treatment modalities initially exhibit efficacy in eradicating *H. pylori*, they frequently give rise to notable adverse effects during the course of administration, including emesis, diarrhea, nausea, and perturbations in intestinal microbiota (Bayerdörffer et al., 1995; Ng et al., 2013). As the treatment progresses, *H. pylori* in a significant proportion of patients develop drug resistance, resulting in treatment failure and occasionally recurrence. The emergence of drug resistance poses significant challenges to the treatment of *H. pylori*, necessitating the development of novel anti-*H. pylori* drugs as an urgent priority.

Throughout history, humans have extensively utilized natural plants for medicinal purposes. Traditional Chinese medicine (TCM) encompasses a plethora of medicinal botanical drug s and food-medicine homologous plants that exhibit potential in the treatment of gastrointestinal disorders, including *Syzygium aromaticum* and *Sanguisorba officinalis L.* (Batiha et al., 2020; Zhao et al., 2017). Moreover, TCM and food-medicine homologous plants exhibit predominantly mild medicinal properties, characterized by a multi-targeted therapeutic profile. Consequently, this treatment approach minimizes the likelihood of drug resistance development while presenting negligible toxic side effects. Meanwhile, certain botanical extracts also demonstrate notable anti-*H. pylori* activity. For instance, *Chenopodium ambrosioides L.* exhibited a minimum inhibitory concentration (MIC) of 16 μg/mL (Ye et al., 2015), *Fragaria vesca* displayed an MIC range of 5–12.5 mg/mL (Cardoso et al., 2018), *Avocados* showed an MIC range of 128–256 μg/mL (Athaydes et al., 2022), and water extracts from *Senna tora (L.) Roxb* and *Fritillaria spp* demonstrated an MIC value of 60 μg/mL (Li et al., 2005). The exploration of natural plant sources for screening bioactive metabolites against *H. pylori* has significant potential in drug discovery.

Triphala was composed of *Terminalia chebula (Terminalia; T. chebula Retz.)*, *Terminalia bellirica (Terminalia; T. bellirica (Gaertn.) Roxb.)*, and *Emblica officinalis (Phyllanthus; Phyllanthus emblica L.)* in a 1:1:1 ratio. As a medicine that has been passed down from ancient times to the present, Triphala is renowned for its nourishing properties, exhibits potent anti-tumor, antibacterial, antioxidant properties, as well as hepatoprotective effects (Vani et al., 1997; Zhao et al., 2017; Omran et al., 2020; Wei et al., 2021). The treatment of gastrointestinal disorders, including gastric ulcer, gastritis, diarrhea, intestinal stress, etc., is accompanied by the inhibitory effect on certain gram-negative bacteria (Tarasiuk, Mosińska, and Fichna, 2018; Mukherjee et al., 2006; Bagde and Sawant, 2013; Khushtar et al., 2016). In our previous screening of ethnic drugs with anti-*H*. *pylori*, we accidently found that Triphala had an inhibitory effect on *H*. *pylori*. However, there is currently a lack of relevant literature elucidating the underlying mechanism responsible for its anti-*H. pylori* activity. Therefore, our research group conducted an investigation to unravel its mode of action against *H. pylori* and its role in combating infections. Our findings demonstrate that it exhibits a potent anti-*H. pylori* effect by effectively inhibiting *H. pylori* survival.

## 2 Materials and methods

### 2.1 Reagents

Brain heart infusion (BHI) and Columbia agar base were purchased from Oxoid Ltd. (Basingstoke, Hants, United Kingdom). Fetal Bovine Serum (FBS) was purchased from Gibco-life Technologies LLC. (Rockville, MD, United States). Sterile-defibrinated sheep blood was purchased from Hongquan Biotechnology Co., Ltd., (Guangzhou, Guangdong, China). Gallic acid (purity: 99%) and Ellagic acid (purity: 96%) were obtained from Aladdin (Shanghai, China). Corilagin (purity: 100%) was obtained from National Institutes for Food and Drug Control, (Beijing, China). Chebulic acid (purity: 99.71%) was bought from Bokang Jingxi Huagong (Shangdong, China). Chebulagic acid (purity: 100%) was bought from PANPH (LOS Angeles, United States). 1% Crystal violet staining solution was bought from Solarbio (Beijing, China). Roswell Park Memorial Institute (RPMI) 1640 medium 1X was obtained from KeyGEN BioTECH (Nanjing, Jiangsu, China). Electron microscope fixative was obtained from Servicebio (Wuhan, Hubei, China). Purelink RNA kit was purchased from Thermo Fisher Scientific (United States). SYBR Premix Ex Tap™ kit and PrimeScript RT reagent kit with gDNA Eraser were purchased from Takara (Minamikusatsu, Japan). DEPC water was purchased from Sangon Biotech (Shanghai, China). BeyoECL Plus Kit, BeyoColor™ Prestained Color Protein Marker, 1% protease inhibitor cocktail, RIPA reagent, BCA protein assay kit, Antifade Mounting Medium, PMSF, SDS-PAGE Gel Preparation Kit, the second antibodies (1:2,500) against mouse (A0216) were obtained from Beyotime (Shanghai, China). PBS 1X was obtained from cytiva. Urea, acetohydroxamic acid, Clarithromycin (CLR), Amoxicillin (AMO) and metronidazole (MET) were purchased from MACKLIN (Shanghai, China). FITC, Tween-20 were purchased from Blotopped (Beijing, China). Ethanol absolute was purchased from Xilong chemical Co., Ltd. (Shantou, Guangdong, China). Phenol red was purchased from Sigma (Germany). Anti-*H. pylori* CagA (sc-28368) was purchased from Santa Cruz Biotechnology (Texas, United States).

### 2.2 *Helicobacter pylori* strains and growth conditions

The standard strains ATCC 43504 and ATCC 700392 were acquired from the American Type Culture Collection (ATCC). The SS1 and CS01 strains were provided by Shanghai Tech University. The clinical strain ICDC111001 was provided by Guangzhou University of Chinese Medicine, while QYZ001, QYZ-003, and QYZ-004 were obtained from Qingyuan Hospital of Traditional Chinese Medicine. The strains were initially identified by the provider based on morphological observation and biochemical reactions. Subsequently, they were preserved at −80°C in a solution containing 65% BHI, 25% glycerol, and 10% FBS (v/v/v). In this study, the strains were cryopreserved, revived, and subcultured on Columbia agar plates supplemented with 5% sterile defibrinated sheep blood. Liquid cultures were conducted in BHI broth containing 10% FBS and incubated at 37°C under a tri-gas mixture of 5% O_2_, 10% CO_2_, and 85% N_2_ for a duration of 72 h in a specialized tri-gas incubator.

### 2.3 Botanical drug materials and sample preparation

*Terminalia chebula* (lot: 210901) used in the experiment was purchased from Guangzhou Zhining Pharmaceutical Co., Ltd. while *T. bellirica* (lot: 220301), and *Emblica officinalis* (lot: 220301) were obtained from Junyuan Shenxiangshan Chinese Medicine Drinking Tablets Co., Ltd. and have been duly identified. The botanical drug materials were stored in the International Pharmaceutical R and D Center of Lunan Pharmaceutical Group. The dried fruits of *T. chebula, T. bellirica*, and *Emblica officinalis* were finely ground and combined in equal proportions (3.3 g: 3.3 g: 3.3 g), then extracted with 10 times the volume of water at a temperature of 90°C for 1 h. This procedure was repeated thrice. Subsequently, the extract was concentrated, freeze-dried, and stored at −20°C. We obtained 4.8 g of the metabolites The experiment was replicated three times.

### 2.4 Phytochemical analysis

#### 2.4.1 UPLC-MS/MS analysis

The solution of Triphala extract was prepared at a concentration of 0.5 mg/mL and subsequently filtered through a 0.22 μm membrane for sample injection. The qualitative analysis was conducted by UPLC-MS/MS from Thermo Scientific. A YMC Triart C18 column (2.1 mm × 100 mm, 1.9 μm) was used. The column temperature was maintained at 25°C with a flow rate of 0.2 mL/min. Detection was performed at a wavelength of 270 nm using both positive and negative ion modes in ESI analysis. The injection volume was 5 μL. For ESI, the capillary voltage was adjusted to +3500v/-3000v, the auxiliary gas flow rate was 10 Arb and sheath gas flow rate was 50 Arb. The evaporation temperature and ion transfer tube temperature were 300°C and 325°C respectively. Mobile phase A was 0.1% formic acid solution, mobile phase B was acetonitrile. Gradient elution parameters: 0–24 min, 97% A and 3%B; 25–35 min, 70% A and 30% B; 36 50 min, 97% A and 3% B.

#### 2.4.2 High-performance liquid chromatography (HPLC) analysis

The extract of Triphala was analyzed by HPLC with Accalim C 18 (4.6*250 mm, 5 um) column. The mobile phase consisted of 0.1% trifluoroacetic acid as solvent A and acetonitrile as solvent B. The flow rate was set at 1 mL/min with a column temperature maintained at 25°C and detection wavelength at 270 nm. The injection volume was 20 μL. Gradient elution parameters: 0–39 min, 97% A and 3% B; 40 min, 70% A and 30% B; 41–50 min, 97% A and 3% B.

#### 2.4.3 FT-IR analysis

Grind Triphala into a fine powder. Take 2 mg of the powder and blend it with potassium bromide, then compress it into tablets. Measure its infrared absorption spectrum within the range of 4,000–400 cm⁻^1^ (Nicolet is50 FTIR spectrometer, Thermo).

### 2.5 Anti-*Helicobacter pylori* activity assays

#### 2.5.1 Determination of minimum inhibitory concentration (MIC)

The MIC of the drug was determined using the microdilution broth method (Peng et al., 2022). The mature *H. pylori* was harvested in PBS, and the turbidity was adjusted to 1 MCF. Then, dilute 10 times with BHI containing 20% FBS. Subsequently, a serial 2-fold dilution method was used to prepare the Triphala solution of six concentrations (1,280 μg/mL, 640 μg/mL, 320 μg/mL, 160 μg/mL, 80 μg/mL, and 40 μg/mL). For test group, 50 μL of Triphala solution of each concentrations was dispensed into each well of a 96-well plate. Then, bacterial suspension of 50 μL was added to each well and mixed by pipetting. For control group, bacterial suspension of 50 μL was added to each well; For negative control group, BHI broth with equivalent Triphala solution was added to each well; For positive control group, clarithromycin (0.128–0.004 μg/mL) and bacterial suspension of 50 μL was added to each well. Three parallel wells were set up for each group. The plates were incubated at microaerobic conditions (37°C) on a shaker at 150 rpm for a duration of 72 h. MIC values were determined by visually observing clear and transparent wells indicating drug concentration levels. This experiment was repeated three times.

#### 2.5.2 Determination of minimum bactericidal concentration (MBC)

Determine the minimum bactericidal concentration (MBC) by assessing the MIC value (Shen et al., 2021). Pipetted 100 μL of *H. pylori* bacterial suspension from the MIC determination plate (4MIC, 2MIC, MIC, control) and individually inoculated onto Columbia blood agar plates supplemented with 5% sheep blood. Ensure even spreading using a spreader stick and incubated at 37°C under microaerophilic conditions for a duration of 5 days. Counted the colonies to determine the MBC, defined as the concentration that exhibits a reduction in bacterial viability by 99.9% compared to the control group.

#### 2.5.3 Inhibiting kinetics assay

Control group (*H. pylori* bacterial suspension in BHI broth containing 10% FBS), test groups (MIC, 0.5MIC, 0.25MIC) and positive control group (*H. pylori* bacterial suspension in 0.016 μg/mL clarithromycin) were cultured under microaerobic conditions at 150 rpm and 37°C for a duration of 3 days. During the incubation, bacterial suspensions of 100 μL were pipetted at time points of 0, 8, 12, 24, 28, 36, 48, 60 and 72 h respectively to measure absorbance values at 600 nm. This experiment was repeated three times.

#### 2.5.4 Killing kinetics assay

Bacterial suspensions of 100 μL were pipetted at time points of 0, 12, 24, 36, 48, 60 and 72 h from Control group (*H. pylori* bacterial suspension in BHI broth containing 10% FBS) and test groups (8MIC, 4MIC, 2MIC), then dilute using a series of ten-fold dilutions (1:10–1:10^9^) method. Pipette 100 μL of the diluted sample and spread it onto a blood agar plate containing sheep blood at a concentration of 5%. Incubate under microaerophilic conditions at 37°C for 5 days. Count the colonies formed on each plate and express the results as Log10 quantities (CFU/mL).

#### 2.5.5 Reassessment of the synergistic bacteriostatic activity between Triphala and antibiotics

The combination therapy of Triphala with four commonly used antibiotics was evaluated using the microdilution checkerboard method. Mature *H. pylori* cells, grown for 72 h, were collected in PBS and the bacterial suspension was adjusted as described in section “2.5.1” Gradient concentrations of Triphala solution ranging from 40 μg/mL to 1,280 μg/mL were prepared using a two-fold dilution method. Additionally, amoxicillin (AMO) concentrations ranged from 8 μg/mL to 0.25 μg/mL, clarithromycin (CLR) concentrations ranged from 0.128 μg/mL to 0.004 μg/mL, metronidazole (MET) concentrations ranged from 8 μg/mL to 0.25 μg/mL, and levofloxacin (LEF) concentrations ranged from 8 μg/mL to 0.25 μg/mL. Mix Triphala solution, antibiotics, and bacterial liquid in a ratio of 1:1:2 to each well with a total volume of 200 μL. Subsequently, place the prepared plate on a shaker operating at 150 rpm under microaerobic conditions at 37°C for a duration of 72 h. The Fractional Inhibitory Concentration Index (FICI) can be calculated using Equation 1 (Krzyżek, Paluch, and Gościniak, 2020), which determines the interaction between Triphala and antibiotics. The FICI value ≤ 0.5 indicates a synergistic effect, 0.5∼1.0 represents an additive effect (partial synergy), and 1.0 ∼ 4 indicates neutral interactions. Conversely, if the FICI exceeds 4, it signifies an antagonistic effect between Triphala and antibiotics.

$$\text{FICI}=\frac{\text{MIC}\left(\text{Triphala}\;\text{combined}\right)}{\text{MIC}\left(\text{Triphala}\;\text{alone}\right)}+\frac{\text{MIC}\left(\text{Antibiotics}\;\text{combined}\right)}{\text{MIC}\left(\text{Antibiotics}\;\text{alone}\right)}$$ (1)

Equation 1 FICI.

### 2.6 Effect on morphology of *Helicobacter pylori*

The effects of Triphala on the ultrastructure of *H. pylori* were investigated using a scanning electron microscope (SEM). After incubation for 72 h, Mature *H. pylori* were harvested and adjusted to a turbidity of 1 MCF unit. Subsequently, incubated in 10% FBS BHI for 24 h. Afterwards, 1 mL of bacterial suspension was pipetted to 49 mL of Triphala solution (1-2 MIC) and 49 mL of 10% FBS BHI respectively, and then incubated for 12 h. The samples were then centrifuged at 6,000 rpm for 3 min to discard the supernatant and collect the bacteria. After being washed twice with PBS solution, they were fixed overnight at 4°C using an electron microscopy fixative. The samples underwent initial dehydration through a graded ethanol series before being freeze-dried and fixed again. Finally, metal coating was applied to the samples prior to observation under a scanning electron microscope (SU8020, Hitachi, Japan).

### 2.7 Influence on virulence genes by using RT-qPCR

*H. pylori* samples were collected from control group and test group (MIC) after incubation of 12 h. Using the PureLink Total RNA Extraction Kit and follow the manufacturer’s instructions to extract total RNA from *H. pylori*. The entire process was conducted on ice, and the extracted RNA was quantified for its concentration. The cDNA was synthesized using reverse transcriptase and stored at −80°C. Taraka TB Green^®^ reagent and the Thermo Scientific 7500 Fast Real-Time PCR System (a quantitative real-time PCR system) were employed for amplification of DNA sequences, and fluorescence signal detection was used to determine the CT value. The 2^−ΔΔCt^ method was employed for data processing, and the expression levels of various genes in the treatment group were assessed by comparing them to the control group using a reference gene (16S) for normalization. The primer sequences utilized are presented in Table 1.

**TABLE 1:** Identification of metabolites in Triphala was performed using the UPLC-MS/MS method.

Peak No	RT (min)	[M-H]^-^(m/z)	(−)MS/MS(m/z)	Error (ppm)	Molecular weight (g/mole)	Molecular formula	Metabolites	Reference
1	1.32	209.0295	85.02811	−0.95	210.0373	C_6_H_10_O_8_	Mucic acid	Chatterjee, Ul Hoda, and Das, (2024)
2	1.49	191.0191	85.02812	−0.52	192.0269	C_6_H_8_O_7_	Citric acid	Peng et al. (2022)
3	1.84	133.0132	71.01248 115.00240	−3.73	134.021	C_4_H_6_O_5_	Malic acid	Yan et al. (2022)
4	1.96	361.0424	85.02813 209.02966	4.69	362.0502	C_13_H_16_O_10_	2-O-Galloylgalactaric acid	Jiang et al. (2024)
7	3.14	355.0311	205.04994 249.04022 337.02032	0.28	356.0388	C_14_H_12_O_11_	Chebulic acid	Wei et al. (2021)
8	3.95	343.0311	85.02811 191.01888	2.9	344.0389	C_13_H_12_O_11_	Mucic acid 2-O-gTLPate 1,4-lactone	Jiang et al. (2024)
9	5.54	331.0676	169.01326 331.06711	3.31	332.0754	C_13_H_16_O_10_	6-O-GTLPoyl-β-D-glucose	Jiang et al. (2024)
10	7.01	169.0134	125.02317	−1.76	170.0212	C_7_H_6_O_5_	Gallic acid	Yan et al. (2022)
11	8.08	343.0311	85.02810 191.01884	2.9	344.0389	C_13_H_12_O_11_	Mucic acid 5-O-gTLPate 1,4-lactone	Jiang et al. (2024)
12	8.55	331.0676	211.02406 271.04587	3.31	332.0754	C_13_H_16_O_10_	2-O-Galloyl-glucose	Jiang et al. (2024)
13	9.71	343.0312	85.02811 191.01891	3.19	344.039	C_13_H_12_O_11_	Mucic acid 3-O-gTLPate 1,4-lactone	Jiang et al. (2024)
22	16.15	483.0785	169.01321 271.04593 313.05655	2.06	484.0863	C_20_H_20_O_14_	3,6-bis-O-GTLPoyl-glucose	Jiang et al. (2024)
24	18.58	633.073	—	0.32	634.0808	C_27_H_22_O_18_	Corilagin	Peng et al. (2022)
25	19.12	651.0844	325.039	1.53	652.0922	C_27_H_24_O_19_	Chebulanin	Pfundstein et al. (2010)
27	20.38	635.0909	169.01323 465.06769	3.77	636.0987	C_27_H_24_O_18_	1,3,6-Tri-O-Galloylglucose	Wei et al. (2021)
28	22.54	953.0898	476.0412 300.99896	0.21	954.0976	C_41_H_30_O_27_	Chebulagic acid	Long et al. (2023)
29	23.35	787.0991	169.01324 465.06760	0.38	788.1069	C_34_H_28_O_22_	1,2,3,6-Tetragalloylglucose	Long et al. (2023)
30	24.31	300.9988	300.99896	0.99	302.0066	C_14_H_6_O_8_	Ellagic acid	Shen et al. (2021)
31	25.3	955.1092	189.01321 205.04994, 275.01974	4.08	956.117	C_41_H_32_O_27_	Chebulinic acid	Bobasa et al. (2021)

### 2.8 Inhibition of *Helicobacter pylori* urease *in vitro*

Following the method outlined in (Debowski et al., 2017), *H. pylori* 700392 a was collected after 72 h of incubation and adjusted the turbidity to 1 MCF. Control group and test groups (2MIC, 1MIC, 1/2MIC, 40 μg/mL AHA) were cultured in a 6-well plate for 24 h. Afterward, bacterial cells were harvested using cold PBS and their OD 600 nm was standardized to 0.4. Next, 50 μL of bacterial suspension were diluted with 50 μL of buffer solution (25 mM PBS containing 0.2% Tween-20). Pipette 25 μL of the diluted bacterial suspension into 150ul of phenol red solution (250 μM, 25 mM PBS). The mixture was incubated at 37°C for 5 min before adding 75 μL of 0.5 M urea. Finally, the absorbance at 560 nm was measured every 72 s for 30 cycles. The enzyme activity measurement involved calculating the rate of change in absorbance over time and expressing it as a percentage relative to the control strain’s urease activity. All measurements were performed in triplicate, and each experiment was repeated three times.

### 2.9 Anti-adhesion experiment

Following the method outlined in (Xu et al., 2017). In each well of a 24-well plate, GES-1 cells (approximately 2 × 10^5^ cells) were seeded in a humidified CO_2_ incubator at 37°C for 24 h. Subsequently, the culture medium was discarded, and the cells were gently washed three times with PBS. Different drug concentrations (4MIC, 2MIC, 1MIC, 0) were prepared using a RPMI 1640 medium containing 10% FBS and added to the 24-well plate along the wall of the well. The cells were treated for 4 h. For *H. pylori* infection, ATCC 700392 bacterial was suspended in PBS and the turbidity was adjust ed to 0.5 MCF. FICI dye was added at a concentration of 1% (v/v) for staining with a ratio of 1:100 under light-avoiding conditions. Staining was conducted for 1 h within a tri-gas incubator protected from light. Following centrifugation at 5,000 *g* and 4°C for 5 min, the supernatant was discarded, and the *H. pylori* samples were subjected to three washes with PBS (0.05% Tween-20) until unbound FITC was eliminated. The *H. pylori* suspension was prepared in 10% FBS 1640 medium and subsequently co-cultured with GES-1 cells for 1 h. Following this, the cells were washed for three times with PBS to eliminate non-adherent bacteria. *H. pylori* and gastric cells were treated by immersing them in a 4% formaldehyde solution for 20 min. The cells were Stained with DAPI dye at room temperature for 3 min, and then washed for 3 times using PBS. Subsequently, an appropriate amount of anti-fluorescence quencher was added to prevent fluorescence quenching. Images were acquired using an IM-5FLD fluorescence inverted microscope (OPTIKA, Italy) at a magnification of ×10. The FITC fluorescence area/DAPI was quantified utilizing ImageJ and subsequently normalized to the control group.

### 2.10 Inhibit the formation of biofilms

Assessment of biofilm formation was conducted using the crystal violet staining method (Hathroubi, Zerebinski, and Ottemann, 2018; Zou et al., 2022). The bacterial suspension was adjusted to 1 MCF and cultured for 24 h in BHI broth supplemented with 20% FBS. Triphala solutions (4MIC, 2MIC, 1MIC, and 0) was prepared with 8% FBS BHI broth. 500 μL of Triphala solution and 50 μL of bacterial suspension was added into a 24 well plate, then incubated statically in a tri-gas incubator for 72 h. The culture medium was discarded and then the plate was washed with PBS and dried at room temperature and then fixated by adding 500 μL of methanol for 10 min. After discarding methanol and air-drying, 500 μL of 0.1% (wt/vol) crystal violet was added to the well and incubated at room temperature for 10 min. After aspirating the crystal violet solution, the plate was rinsed three times with PBS and dried at room temperature. 500 μL of anhydrous ethanol was added to each well and the absorbance was tested at 560 nm for visualization of biofilm formation. The obtained data was normalized against a control group for growth, and the experiment was replicated thrice.

### 2.11 Experimental investigation of CagA protein expression

Control group and test groups (2MIC, MIC) were collected after incubation for 24 h 120 μL of RIPA lysis buffer containing 1 mM PMSF and 1% proteinase inhibitor was used for *H. pylori* cleavage for each group, then *H. pylori* proteins was collected. The BCA assay kit was used to determine the protein concentrations of each group, enabling accurate quantification of the protein load. 20 μg of proteins were loaded in each group and subsequently separated by SDS-PAGE gel. The separated proteins were then transferred onto a PVDF membrane. Following the transfer, the membrane was blocked using 5% skim milk. The PVDF membrane was incubate overnight with the primary antibody at 4°C, followed by subsequent incubation with the corresponding secondary antibody. The bands were exposed and imaged through BeyoECL kit (Beyotime, China) and the fully automated chemiluminescence imager (ChemScope 6200, Clinx Science Instruments). Grayscale analysis was Performed by the ImageJ software.

### 2.12 Statistical analysis

Data analysis was conducted using GraphPad Prism 8.0.2 software, and statistical analyses were performed using either a two-tailed Student’s t-test or non-parametric tests, followed by *post hoc* testing. The observed difference exhibits statistical significance at a level of p < 0.05.

## 3 Result

### 3.1 UHPLC-MS/MS and HPLC

**FIGURE 1:**
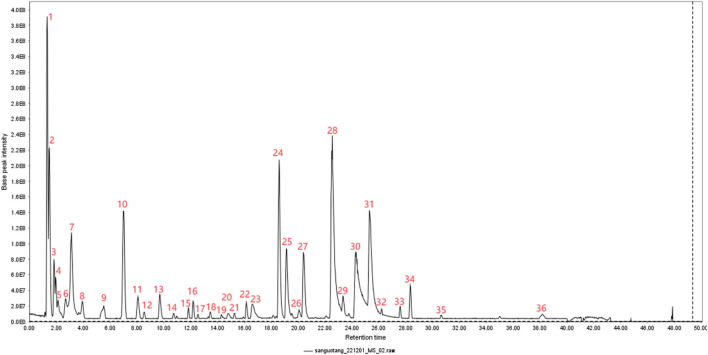
Plot of total TIC of extracts of Triphala.

**FIGURE 2:**
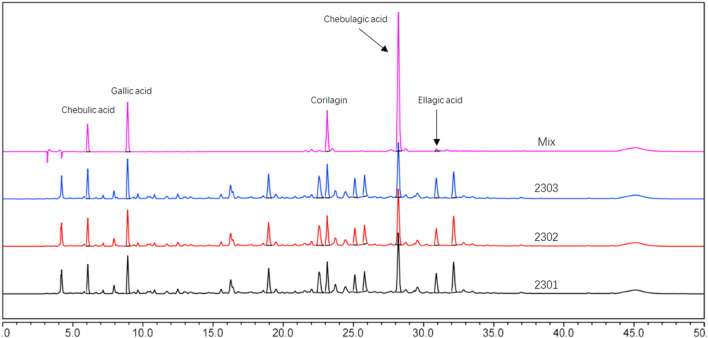
HPLC chromatograms of three batches of Triphala extracts.

In this experiment, the metabolites of the extracts from Triphala were analyzed using ultra-high performance liquid chromatography-tandem mass spectrometry (UHPLC-MS/MS). The total ion chromatogram is shown in Figure 1, and a total of 19 major metabolites were identified and listed in Table 1. To ensure the stability of the metabolites in the extract of Triphala, a quantitative analysis was performed using HPLC on three batches (Figure 2). Quantitative results are listed in Table 2, while linear variance and range can be found in Table 3. The findings demonstrate consistent levels of the five primary metabolites, with Chebulagic acid exhibiting the highest concentration.

**TABLE 2:** Content of active metabolites in Triphala.

NO	Chebulic acid	Gallic acid	Corilagin	Chebulagic acid	Ellagic acid
2301	3.4%	1.4%	2.6%	6.8%	0.7%
2302	3.3%	1.4%	2.5%	6.4%	0.6%
2304	3.5%	1.5%	2.8%	6.2%	0.7%

**TABLE 3:** Linear range data of effective metabolites in Triphala.

Metabolites	Regression equation	R^2^	Range (μg/mL)
Chebulic acid	A = 18.95°C + 8.21	1.00	4.0442–15.1659
Gallic acid	A = 64.50°C − 1.31	1.00	2.2988–8.6204
Corilagin	A = 35.54°C + 3.61	0.99	4.032–15.120
Chebulagic acid	A = 27.24°C + 44.35	1.00	19.00–75.00
Ellagic acid	A = 107.65°C − 22.36	1.00	1.1952–4.482

### 3.2 FT-IR

**FIGURE 3:**
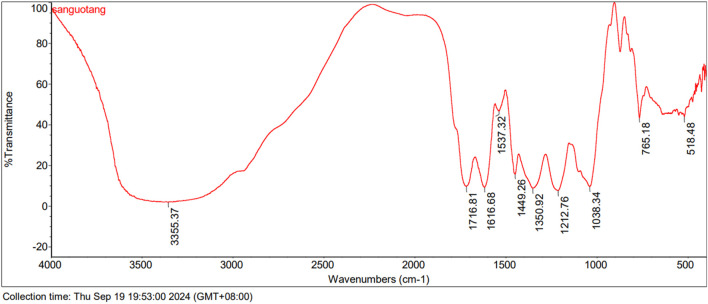
FTIR spectra of Triphala.

The results show that Triphala contains a diverse range of tannin metabolites characterized by α,β-unsaturated ester bonds, glycosidic linkages, and aromatic rings (Ahmed, Ding, and Sharma, 2021). The characteristic spectral peaks are: 3355.37 cm⁻^1^ for O-H stretching; 1716.81 cm⁻^1^ for α,β-unsaturated esters; 1616.68 cm⁻^1^, 1537.32 cm⁻^1^, and 1449.26 cm⁻^1^ for benzene ring vibrations; 1350.92 cm⁻^1^ for carboxylate (COO⁻) groups; and 1212.76 cm⁻^1^ and 1038.34 cm⁻^1^ for C-O stretching (Figure 3).

### 3.3 Result of MIC and MBC

The MIC values were determined using a 96-well plate, as listed in Table 4. Triphala exhibited consistent MIC values across multiple strains, ranging from 80 to 320 μg/mL. However, the MBC varies among different strains. The MBCs of ATCC 43504, ATCC 700392, and ICDC 111001 clinical strains are all within a range of 1–2 times the MIC. Triphala exhibits potent bactericidal activity and is regarded as an effective bactericide. Generally, the MBC within 4 times the MIC is considered as indicative of bactericidal activity, while MBC values exceeding 4MIC are typically associated with bacteriostatic effects. In the case of clinical strains QYZ-003 and CSO1, Triphala demonstrates a bacteriostatic effect with an MBC value greater than 4MIC, thus classifying it as a bacteriostatic agent.

**TABLE 4:** The results of MIC and MBC for Triphala.

Drugs	*H.pylori* strains	Drug sensitivity	MIC (μg/mL)	MBC (μg/mL)	MBC/MIC	CLR (μg/mL)
Triphala	ATCC 43504	R (MTZ)	160	160–320	1–2	0.016
	ATCC 700392	S	160	320	2	0.004
	ICDC 111001	R (MTZ, LEF)	160	320	2	>0.128
	QYZ-003	R (CLR, MTZ, LEF)	80	>320	>4	>0.128
	CSO1	R (CLR)	320	>1,280	>4	>0.128

Note: S, drug sensitive; R, drug resistant.

### 3.4 Inhibiting kinetics assay and killing kinetics assay

**FIGURE 4:**
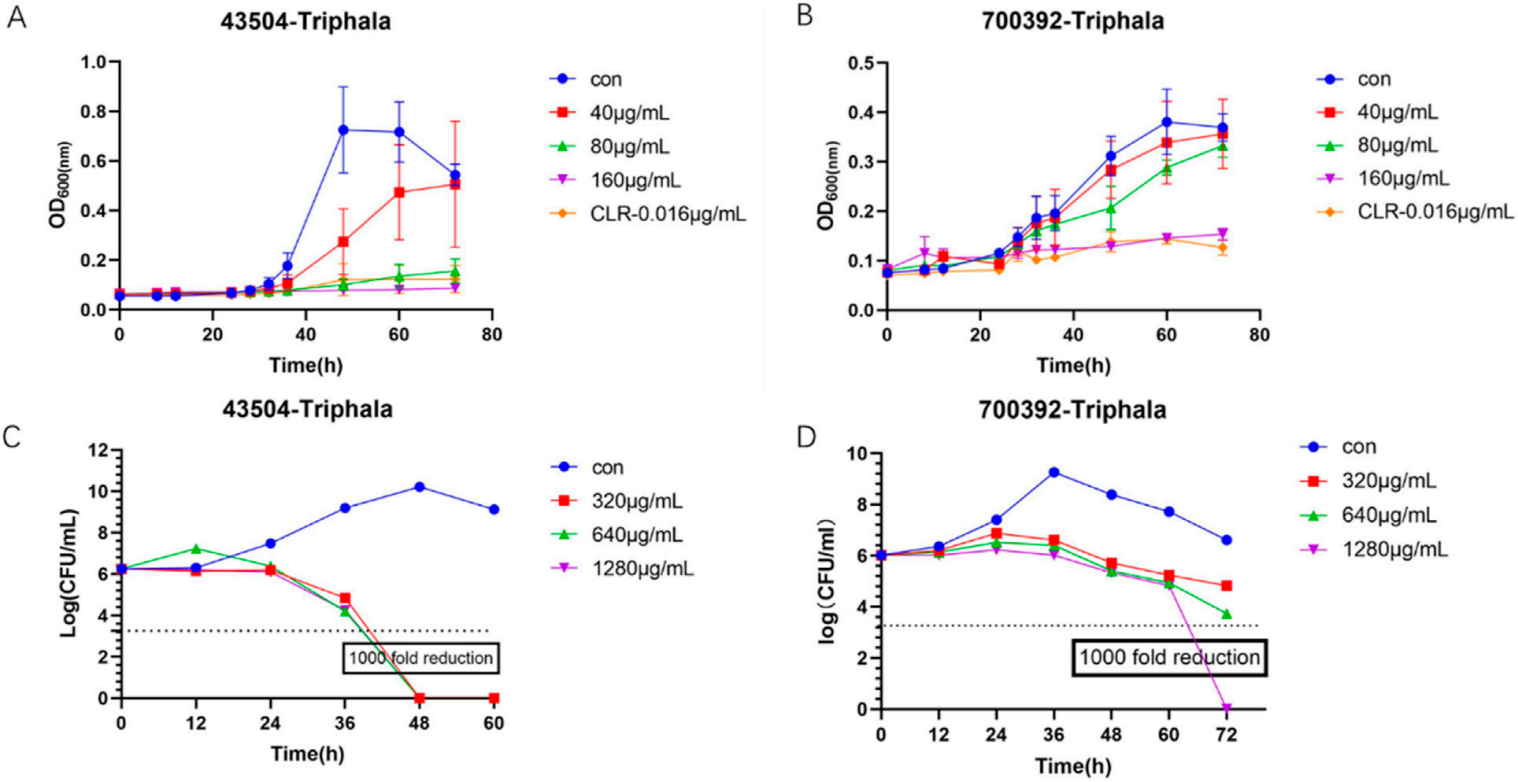
Bacteriostatic Curve and Bactericidal Curve. **(A)** Time-dependent antibacterial activity of Triphala against ATCC 43504. **(B)** Time-dependent antibacterial activity of Triphala against ATCC 700392. **(C)** Time-dependent bactericidal effect of Triphala against ATCC 43504. **(D)** Time-dependent bactericidal effect of Triphala against ATCC 700392. Con = Control.

The experimental results are listed in Figure 4. The antibacterial kinetics of Triphala against ATCC 43504 and ATCC 70039 were assessed, revealing that Triphala exhibited superior antibacterial efficacy against *H. pylori* at the MIC level. It effectively inhibited bacterial growth within 72 h, even at concentrations ranging from 1/4MIC to 1/2MIC, thus demonstrating promising antibacterial effects. Notably, the observed antibacterial efficacy displayed a dose-dependent increase. In the bactericidal curve of *H. pylori* by treatment with Triphala, a significant reduction in *H. pylori* count was observed after 5 days of incubation at a dosage of 2–4 times the MIC, resulting in more than a thousand-fold decrease compared to the initial inoculum. Remarkably, Triphala exhibited potent bactericidal activity against *H. pylori* within 48 h.

### 3.5 Combined antibacterial results

The results listed in Table 5 demonstrate the absence of any antagonistic effect when Triphala is combined with four antibiotics, instead revealing an neutral effect.

**TABLE 5:** Results of combination therapy.

Antibiotic	*H.pylori* strains	MIC (μg/mL)			FICI
		Triphala	Antibiotic	Triphala+Antibiotic	
CLR	ATCC 700392	160	0.004	160 + 0.001/0.004 + 40	1.250
AMO	ATCC 700392	160	0.125	160 + 0.03125/0.0125 + 40	1.250
LEF	ATCC 700392	160	1	160 + 0.125/1 + 20	1.125
MET	ATCC 700392	160	2	160 + 0.5/2 + 40	1.250

### 3.6 Effect of Triphala on the microscopic morphology of *Helicobacter pylori*

**FIGURE 5:**
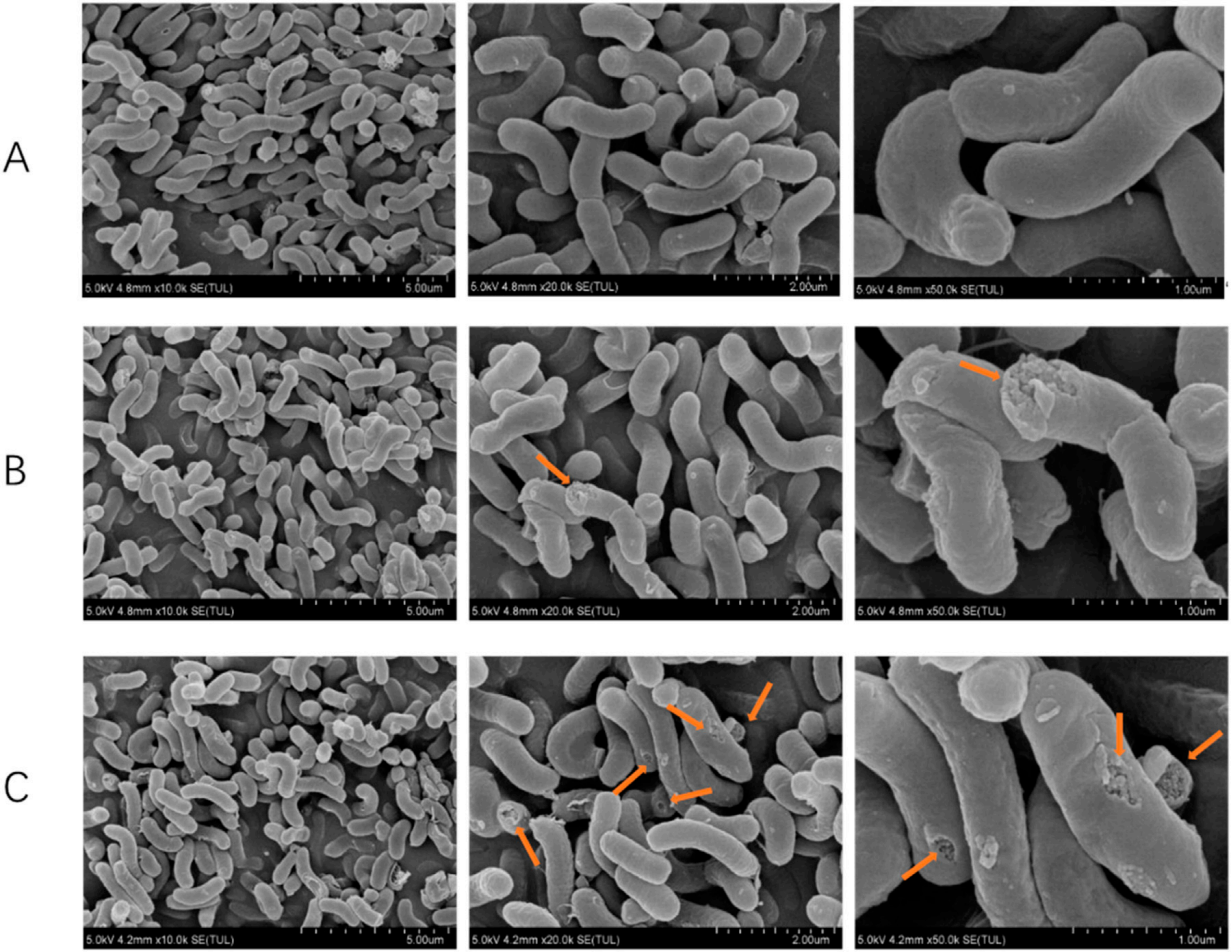
Scanning electron microscope rendering of *Helicobacter pylori*. **(A)** Control group, **(B)** Triphala (MIC), **(C)** Triphala (2MIC).

The experimental results are listed in Figure 5, illustrating the observation and analysis of the ultrastructure of *H. pylori* treated with 1-2MIC Triphala. Under electron microscopy, the control group of bacteria exhibited a smooth and intact surface, predominantly appearing as spiral rods. In contrast, the *H. pylori*-treated group displayed evident damage and shrinkage on both its surface and head, indicating a dose-dependent destructive impact on *H. pylori*.

### 3.7 RT-qPCR results

**FIGURE 6:**
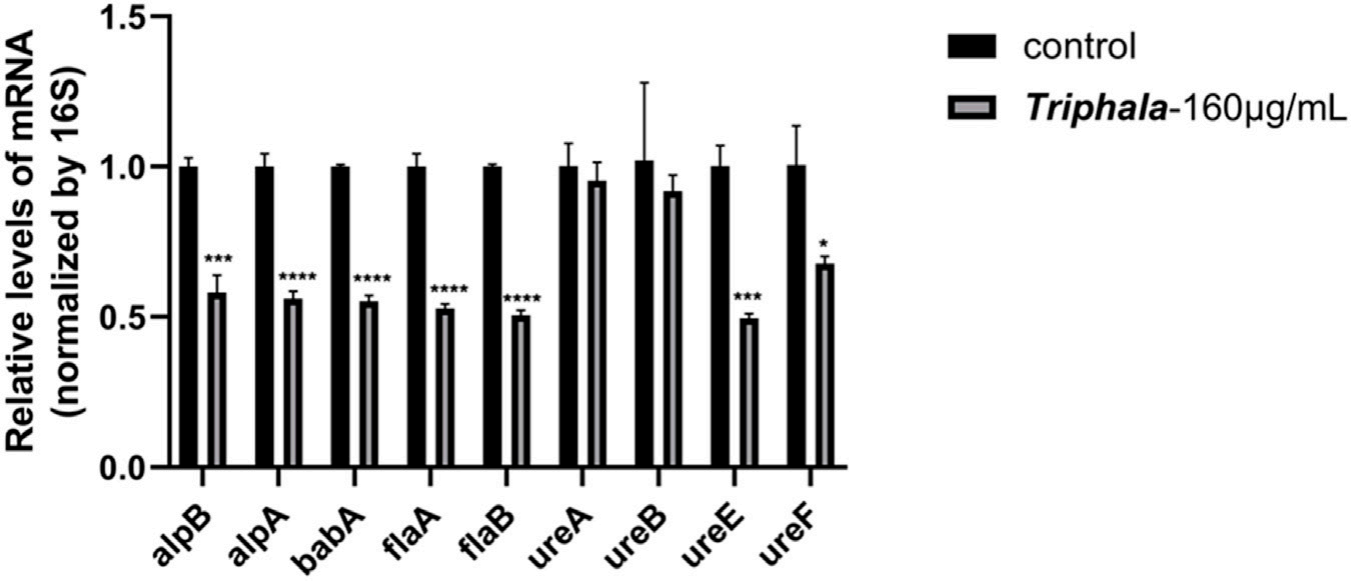
Effects of Triphala on the expression of various virulence factors in *Helicobacter pylori*. Test groups VS control group, *P < 0.05, **P < 0.01, ***P < 0.001, ****P < 0.0001.

The experimental results are listed in Figure 6, and the virulence factor within *H. pylori* is commonly regarded as a pivotal gene associated with *H. pylori* infection. The results demonstrated that, in comparison with the control group, treatment with Triphala at MIC concentration for 12 h significantly downregulated the expression of adhesin genes *alpA*, *alpB*, *babA*, flagellar genes *flaA* and *flaB*, as well as urease genes *ureE* and *ureF* by Triphala. Moreover, it exhibited a certain inhibitory effect on urease genes *ureA* and *ureB*.

### 3.8 Anti-adhesion results

**FIGURE 7:**
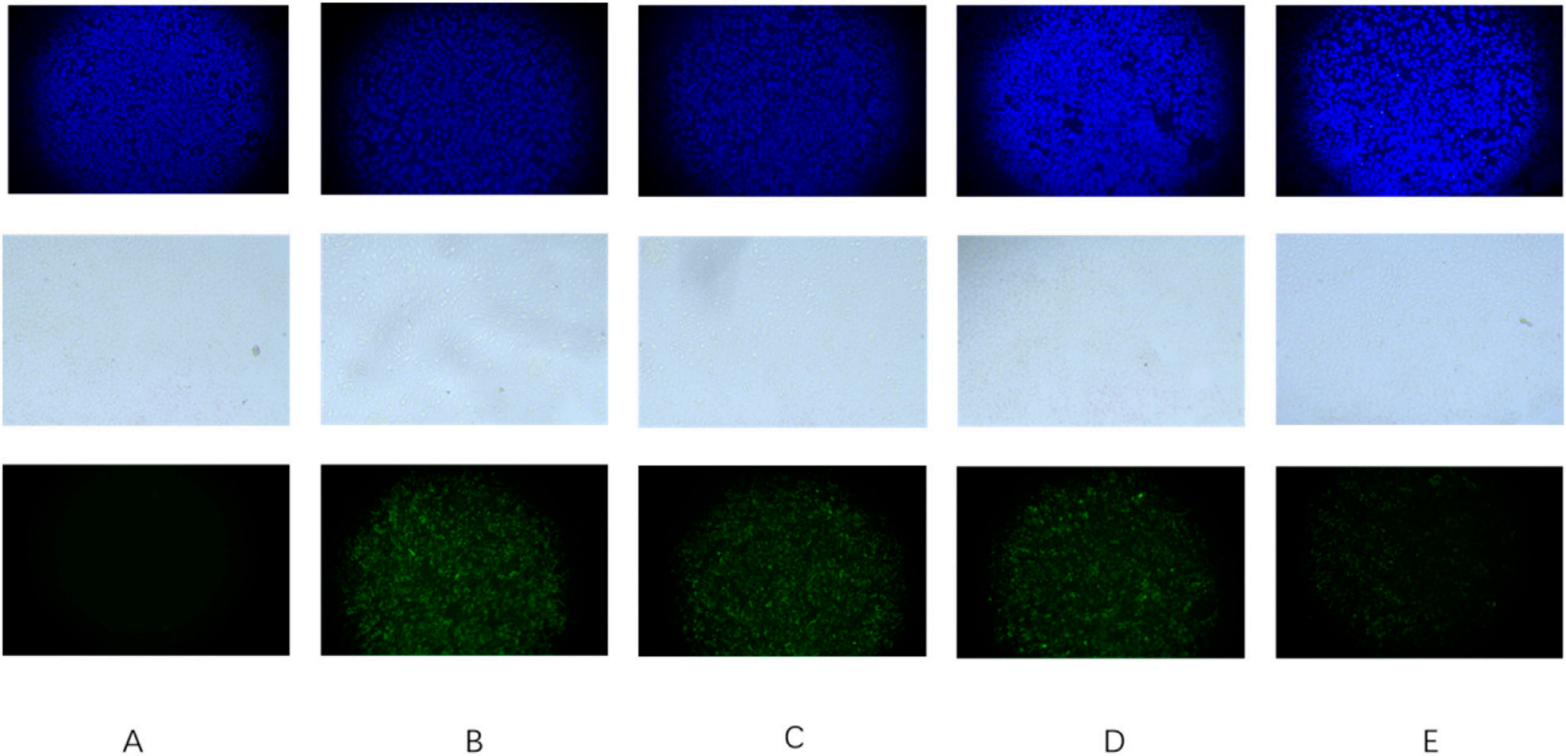
Triphala prevents *Helicobacter pylori* adhesion to GES-1 cells. **(A)** Control group of cells, **(B)** Cells + ATCC 700392, **(C)** Cells treated with Triphala (MIC) + ATCC 700392, **(D)** Cells treated with Triphala (2MIC) + ATCC 700392, and **(E)** Cells treated with Triphala (4MIC) + ATCC 700392. Bar = 50 μm. The blue fluorescent image represents a cellular image stained with DAPI, while the white image depicts cells under bright field illumination. Additionally, the green fluorescent image displays an Hp stain using FITC.

**FIGURE 8:**
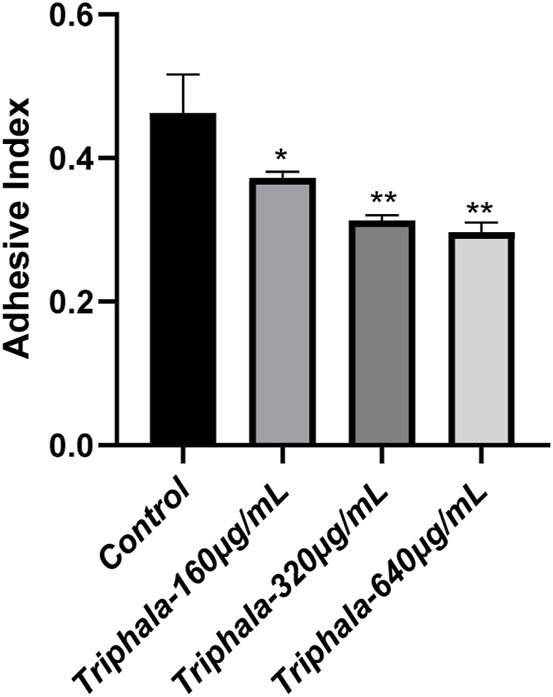
Adhesion Index of *Helicobacter pylori*. test groups VS control group, *P < 0.05, **P < 0.01.

The findings from the extracellular anti-adhesion experiment are listed in Figures 7, 8. After treatment with Triphala in GES-1 cells, the adhesion efficiency of *H. pylori* decreased compared to the control group, and a dose-dependent increase in the inhibitory effect on adhesion was observed. This phenomenon may be attributed to the multi-target mechanism of action exhibited by traditional Chinese medicine. Specifically, Triphala is hypothesized to bind and obstruct specific binding proteins on GES-1 cells, thereby competitively inhibiting *H. pylori*-GES-1 cell binding and adhesion, ultimately demonstrating a preventive effect (Chen et al., 2022).

### 3.9 Urease activity results

**FIGURE 9:**
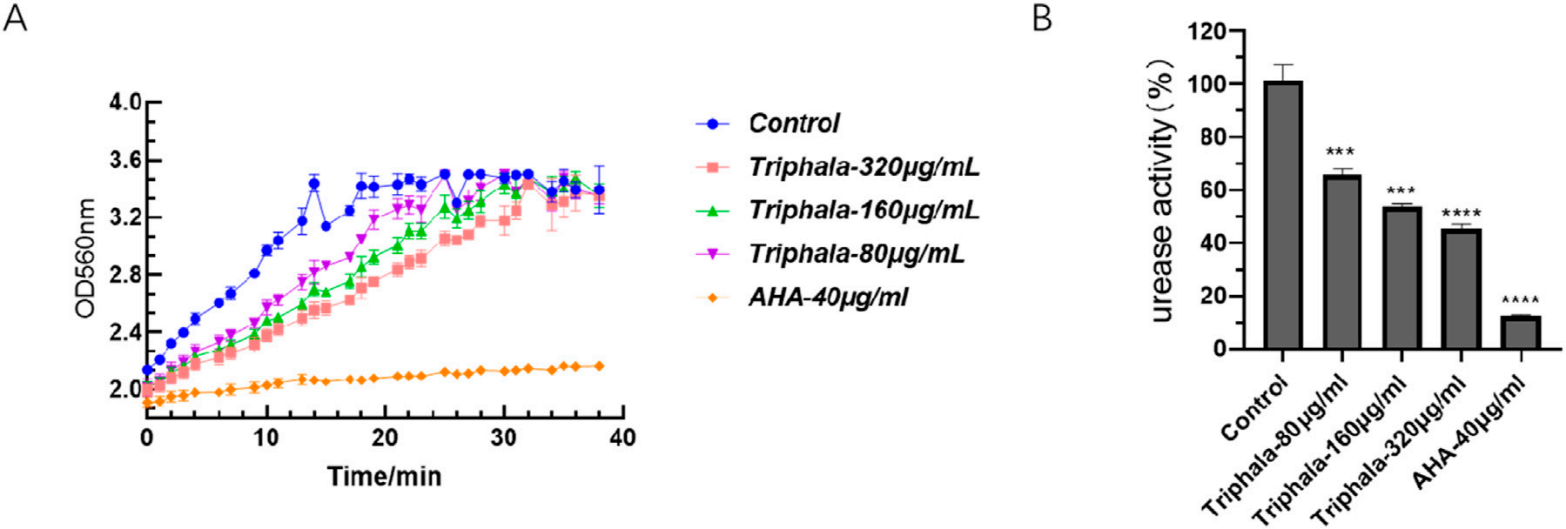
Inhibition of Urease Activity. **(A)** Absorption kinetics curve of urease activity at 560 nm for ATCC 700392. **(B)** Normalized urease activity of ATCC 700392 after normalization by the control group. test groups vs. control group, *P < 0.05, **P < 0.01, ***P < 0.001, ****P < 0.0001.

The results of the extracellular urease experiment are presented in Figure 9. Following 24 h of drug treatment, the absorption curve of the treatment group exhibits a delayed attainment of plateau phase compared to the control group. The rate of absorption change in the treatment group demonstrates a dose-dependent reduction effect, with higher drug concentrations resulting in diminished alterations in the curve. These findings suggest a concurrent decline in urease activity. Triphala exerts significant inhibitory effects on urease activity.

### 3.10 Inhibit the formation of biofilm

**FIGURE 10:**
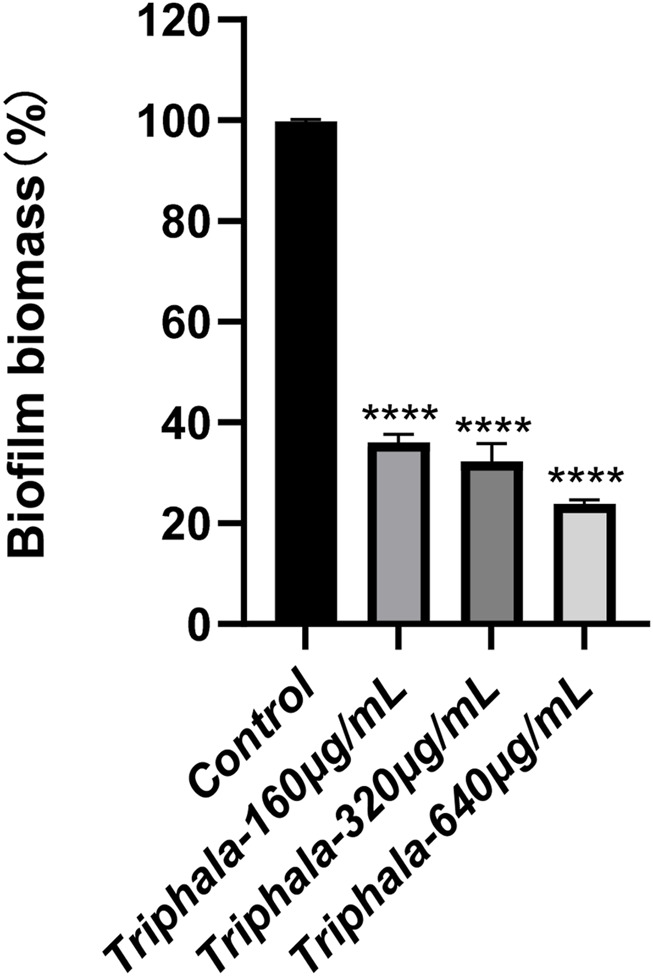
Inhibitory effect of Triphala on the biomass of *Helicobacter pylori* biofilm. Test groups VS control group, *P < 0.05, **P < 0.01, ***P < 0.001, ****P < 0.0001.

The experimental results listed in Figure 10 demonstrate the effects of crystal violet staining on biofilms. Triphala was administered at concentrations of 160 μg/mL, 320 μg/mL, and 640 μg/mL for a duration of 72 h. In comparison to the control group, test groups significantly impeded biofilm formation and induced fragmentation of *H. pylori* biofilms. This disruption prevented *H. pylori* from forming intact biofilms as a defense mechanism against drug invasion, with the inhibitory effect exhibiting a dose-dependent escalation.

### 3.11 Inhibition of CagA protein expression

**FIGURE 11:**
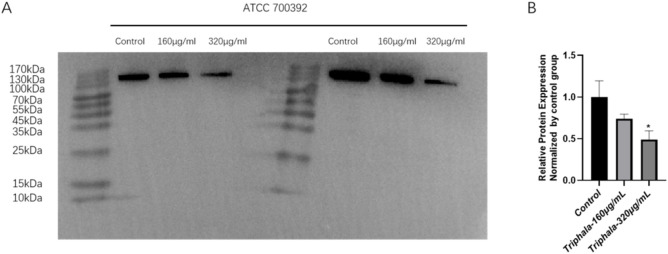
Inhibits the expression of CagA protein by Triphala. **(A)** Fluorescent band membrane of the target protein after 24 h of treatment with ATCC 700392 was observed. **(B)** Comparison of target protein expression levels between the ATCC 700392. test groups VS control group, *P < 0.05.

The results of the Western blot experiment are listed in Figure 11. Treatment with Triphala at concentrations of 160 μg/mL and 320 μg/mL for a duration of 24 h significantly induced a dose-dependent decrease in the expression level of CagA protein, demonstrating notable inhibitory effects.

## 4 Discussion

The present study conducts a comprehensive metabolite analysis of the Triphala with demonstrated anti-*H. pylori* activity, further elucidating and unveiling its mechanism of action *in vitro* against *H. pylori*.

We have selected the microdilution broth method to determine the MIC. The MIC values of Triphala against both standard and various clinical strains ranged from 80 to 320 μg/mL, while the MBC values for standard strains were found to be within 1–2 times the MIC, indicating its potent bactericidal effect. It is noteworthy that Triphala exhibits differential bactericidal effects on various clinical strains, acting as a bactericidal agent against ICDC 111001 while demonstrating bacteriostatic activity against clinical strains of this bacterium. This discrepancy may be attributed to subtle and unidentified variations among the clinical strains.

The combination therapy experiments demonstrated that Triphala exhibited favorable compatibility with four distinct antibiotics, devoid of any antagonistic effects. Consequently, it can be employed as an adjunct to antibiotic treatment for *H. pylori* infection, thereby mitigating the discomfort and side effects associated with sole antibiotic administration.

Utilizing a scanning electron microscope for the observation of morphological alterations induced by drugs on bacteria can visually exemplify the deleterious impact of drugs on *H. pylori*’s appearance, thereby further elucidating their inhibitory and bactericidal effects against *H. pylori*. Based on the antibacterial curve results, it can be inferred that a 12-h administration of Triphala does not significantly affect the logarithmic growth cycle of bacteria, thereby eliminating potential interference from bacterial quantity in the experiment. Under the influence of antibacterial drugs, bacteria undergo structural damage, resulting in the appearance of wrinkled and compromised bacterial bodies. This phenomenon leads to intracellular substance leakage and ultimately culminates in bacterial death. Moreover, this destructive effect exhibits a dose-dependent relationship, with higher doses causing more pronounced destruction of bacterial bodies.

The infectivity of *H. pylori* is commonly believed to be closely associated with its virulence factors within the host, encompassing flagellar, adhesion, urease genes, as well as VacA and CagA genes. *H. pylori* exhibits flagella-driven motility in gastric fluid, facilitating its penetration through the mucus layer and colonization of surface epithelial cells. Additionally, it secretes adhesins to firmly adhere to GES-1 cells (Johnson and Ottemann, 2018). *flaA* and *flaB* are the principal constituents of *H. pylori* flagella, while *alpA* and *alpB* contribute to *H. pylori* colonization and adhesion in conjunction with *babA* (Josenhans, Labigne, and Suerbaum, 1995; Borén et al., 1993; Senkovich et al., 2011) The urease enzyme of the organism continuously hydrolyzes urea and buffers gastric acid, enabling its survival under favorable conditions (Eaton et al., 1991). *ureA* and *ureB* serve as structural components of urease, while the accessory proteins *ureE* and *ureF* play pivotal roles in facilitating urease activation through nickel ion transportation and removal of non-carbamylated protein binding (Li et al., 2019). In the presence of antibacterial concentration, Triphala significantly downregulates the expression of virulence factors, including *alpA*, *alpB*, *babA*, *flaA*, *flaB*, *ureE*, and *ureF*. Furthermore, it exerts a discernible inhibitory effect on *ureA* and *ureB*. The characterization experiment demonstrated a significant inhibitory effect of Triphala on urease activity, as well as its ability to significantly inhibit the formation of biofilms, which is believed to be closely related to *alpB* and flagellar genes. These results were consistent with those obtained from RT-qPCR analysis, providing further insight into the antibacterial mechanism of Triphala.

The adherence of *H. pylori* to the gastric environment is robust, ensuring its resistance against washout during stomach emptying. Bacterial adhesion serves as a pivotal foundation and prerequisite for cellular infection and the generation of toxic effects. The results of the anti-adhesion test demonstrated a significant reduction in *H. pylori* adhesion quantity after administration to GES-1 cells, with the decreasing effect on adhesion quantity exhibiting a dose-dependent increase. It is hypothesized that Triphala may exert a preventive effect on *H. pylori* adhesion by inhibiting the receptor associated with adhesion in GES-1 cells, thereby impeding *H. pylori*-cell interaction.

CagA, also known as cytotoxin-associated gene A, is a 120–130 Kda protein encoded by cagPAI. *H. pylori* delivers CagA to host cells via the Cag type IV secretion system (T4SS), where it becomes phosphorylated and activates SHP-2 and PI3K/Akt signaling pathways, promoting inflammation and carcinogenesis (Coticchia et al., 2006; Censini et al., 1996). The experimental results demonstrate that Triphala effectively downregulates the expression level of CagA protein, exhibiting a clear dose-dependent response.

## 5 Conclusion

In conclusion, research on the extracellular antibacterial effects of Triphala has substantiated its inhibitory and bactericidal efficacy against drug-resistant sensitive bacteria as well as standard strains. Triphala exerts a disruptive effect on the microstructure of *H. pylori*, thereby impacting bacterial survival and significantly suppressing the expression of diverse virulence factors within the bacteria. We further validated the inhibitory effect of Triphala on urease activity through phenotypic experiments, which is consistent with the results obtained from RT-qPCR. The above experiment provides a preliminary elucidation of the mechanism and efficacy of Triphala in combating *H. pylori*, necessitating further investigation into its underlying mechanisms. Meanwhile, it also demonstrates the potential of Triphala as a medicine with anti-*H. pylori* properties.

## Data Availability

The raw data supporting the conclusions of this article will be made available by the authors upon request.
